# Pulmonary Vein Stenosis Complicating Radiofrequency Catheter Ablation: Five Case Reports and Literature Review

**DOI:** 10.1097/MD.0000000000001346

**Published:** 2015-08-28

**Authors:** Hai-Wen Lu, Ping Wei, Sen Jiang, Shu-yi Gu, Li-Chao Fan, Shuo liang, Xiaobin Ji, Bhavana Rajbanshi, Jin-Fu Xu

**Affiliations:** From the Department of Respiratory Medicine, Shanghai Pulmonary Hospital, Tongji University School of Medicine, Shanghai, China.

## Abstract

The aim of this study is to characterize the clinical manifestations and features of pulmonary vein stenosis (PVS) by retrospectively analyzing clinical data of patients in addition to reviewing the literature simultaneously to improve the understanding of PVS complicating radiofrequency catheter ablation and to provide evidence for early diagnosis and timely treatment.

Clinical, imaging, and follow-up data of 5 patients with PVS-complicating radiofrequency catheter ablation were retrospectively analyzed between January 2012 and December 2014 in Shanghai Pulmonary Hospital, Tongji University School of Medicine, Shanghai, China. Relevant studies previously reported were also reviewed.

Three out of 5 patients received pulmonary angiography. The initial symptoms were not specific, presenting chest pain in 3 cases, hemoptysis in 2 cases. The average duration between radiofrequency ablation to the onset of symptoms was 5.8 months. The chest image results were consolidation and pleural effusion mainly. Veins distributed in the left lungs were mostly influenced in 4 patients, and the inferior veins in 3 patients. Cardiac ultrasound examinations showed pulmonary arterial hypertension in 2 patients. Two patients received selective bronchial artery embolization after bronchial artery radiography because of hemoptysis. One patient underwent video-assisted thoracoscopic biopsy because of the suspicion of tumor.

PVS is a condition mostly undetected because of its silent manifestations and inconsistent follow-up. The accurate clinical diagnosis is very difficult. A careful review of medical history and follow-up observation may be useful for all the patients who received the radiofrequency catheter ablation to recognize PVS in the early stage.

## INTRODUCTION

Radiofrequency catheter ablation was firstly introduced into clinical practice in 1987, which has now become an effective treatment for atrial fibrillation (AF). Since then, this treatment method has been widely applied. Despite the successes, pulmonary vein stenosis (PVS) is one of the most serious complications for patients receiving radiofrequency catheter ablation.

Some patients with isolated mild PVS are asymptomatic, while patients with more extensive and severe involvement may manifest dyspnea, cough, chest pain, and hemoptysis.^1–4^ Most of the patients are therefore seeking medical care from the Department of Respiratory Medicine and can be easily misdiagnosed as pulmonary embolism (PE), pneumonia, or tuberculosis. Hence, the present work aimed to characterize the clinical manifestations and features of PSV by retrospectively analyzing the clinical data in addition to simultaneously reviewing the articles published.

## MATERIALS AND METHODS

### Subjects and Study Design

Clinical data of patients with PVS-complicating radiofrequency catheter ablation, admitted to Shanghai Pulmonary Hospital, Tongji University School of Medicine Shanghai, China, between January 2012 and December 2014, were retrospectively analyzed in this case series. Five patients were diagnosed with PVS on the basis of multislice spiral chest computed tomography (CT), and 3 of them accepted pulmonary angiography. The last follow-up was from initial diagnosis to January 31, 2015. The study protocol was approved by the Ethics Committee of the Shanghai Pulmonary Hospital. The patient consent was waived because the study is a retrospective one.

### Laboratory Tests

All patients underwent routine peripheral blood cell tests, D-D dimer, and arterial blood gas analysis. Culture tests for pathogenic microorganisms from sputum and bronchoalveolar lavage fluid were also performed.

### Imaging Examination

Examinations including chest X-ray, multislice spiral chest CT angiography, echocardiography, pulmonary perfusion ventilation imaging, and pulmonary angiography were done.

## RESULTS

### Baseline and Demographic Information

A retrospective analysis identified 5 patients with PVS-complicating radiofrequency catheter ablation who were discharged from the hospital. The case series included 4 male and 1 female patients of Chinese Han race, aged between 53 and 64 years (57.4 years in average).

All 5 patients received radiofrequency catheter ablation within the pulmonary veins because of AF. One patient received point-by-point ablation (the melting power was 30 W). Four cases received 1 AF ablation procedure. One patient (Case 2) received 2 AF ablation procedures (one 4 years before and another one half year before the symptoms occurred). All the patients received oral Warfarin anticoagulation therapy immediately following their radiofrequency catheter ablation procedure. The courses ranged from 3 to 6 months.

In this group, all the patients had a variety of symptoms, including chest pain (4 cases), shortness of breath (3 cases), gross hemoptysis (3 cases), minimum blood streaking in the sputum (2 cases), productive of cough (2 cases), and fever (1 case). All of the patients had 1 or more of the aforementioned symptoms, but only 1 patient's symptoms were very serious. The median duration between radiofrequency ablation to the onset of respiratory symptoms of patients was 5.8 months. Physical examination indicated that the accentuation of the second heart sound in the pulmonary valve auscultation area was detected in 1 patient (Table 1).

**TABLE 1:**
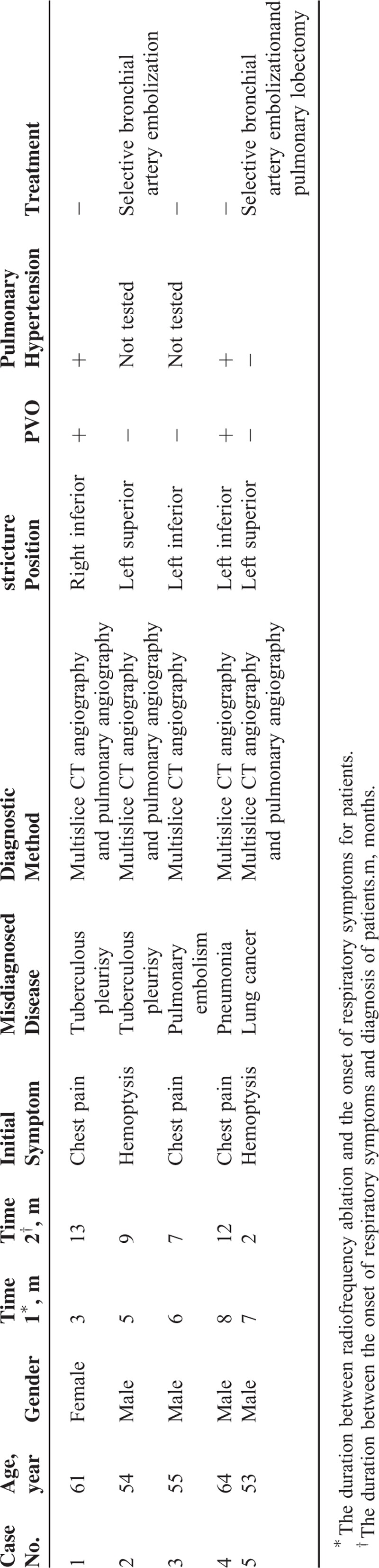
Clinical Information for 5 PVS Patients

### Laboratory Tests

All the patients received peripheral blood cell tests, D-D dimer, and arterial blood gas analysis. Three patients presented hypoxemia. The D-D dimer of 2 patients increased slightly. Pathogenic microorganism's cultures from the sputum and bronchoalveolar lavage fluid were all negative (Table 2).

**TABLE 2:**
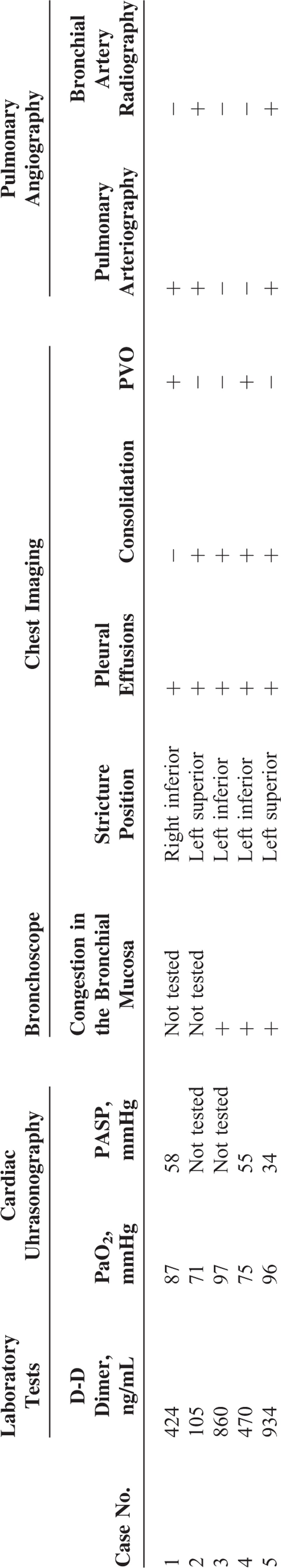
Laboratory Testing for 5 PSV Patients

### Radiographic Findings on the Chest Radiograph

**FIGURE 1:**
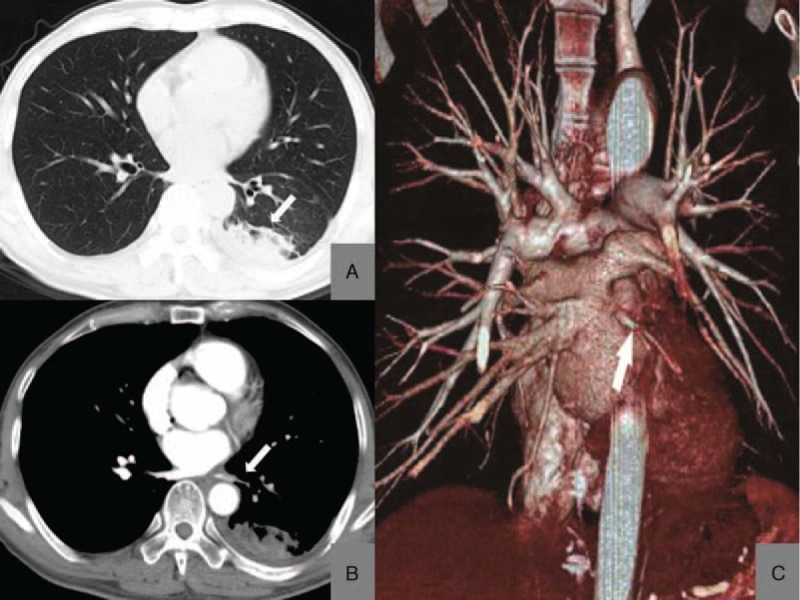
Multislice CT scan for PVS patient. (A, B) Case 3: CT shows little consolidation shadows and pleural effusion in the left lung. (C) Case 3: 3D CT angiogram shows of left inferior pulmonary veins. CT = computed tomography, PVS = pulmonary vein stenosis.

All the patients received the multislice spiral CT angiography and details were shown in Table 2. The majority chest radiographic findings of PVS include consolidation and pleural effusion. Consolidation was detected in 4 cases, whereas all the chest radiographs showed pleural effusion. Veins distributed in the left lung were mostly influenced in 4 patients, and the inferior veins in 3 patients (Figure 1 from Case 3).

### The Ventilation (V)/Perfusion (Q) Scan (V/Q Scan)

**FIGURE 2:**
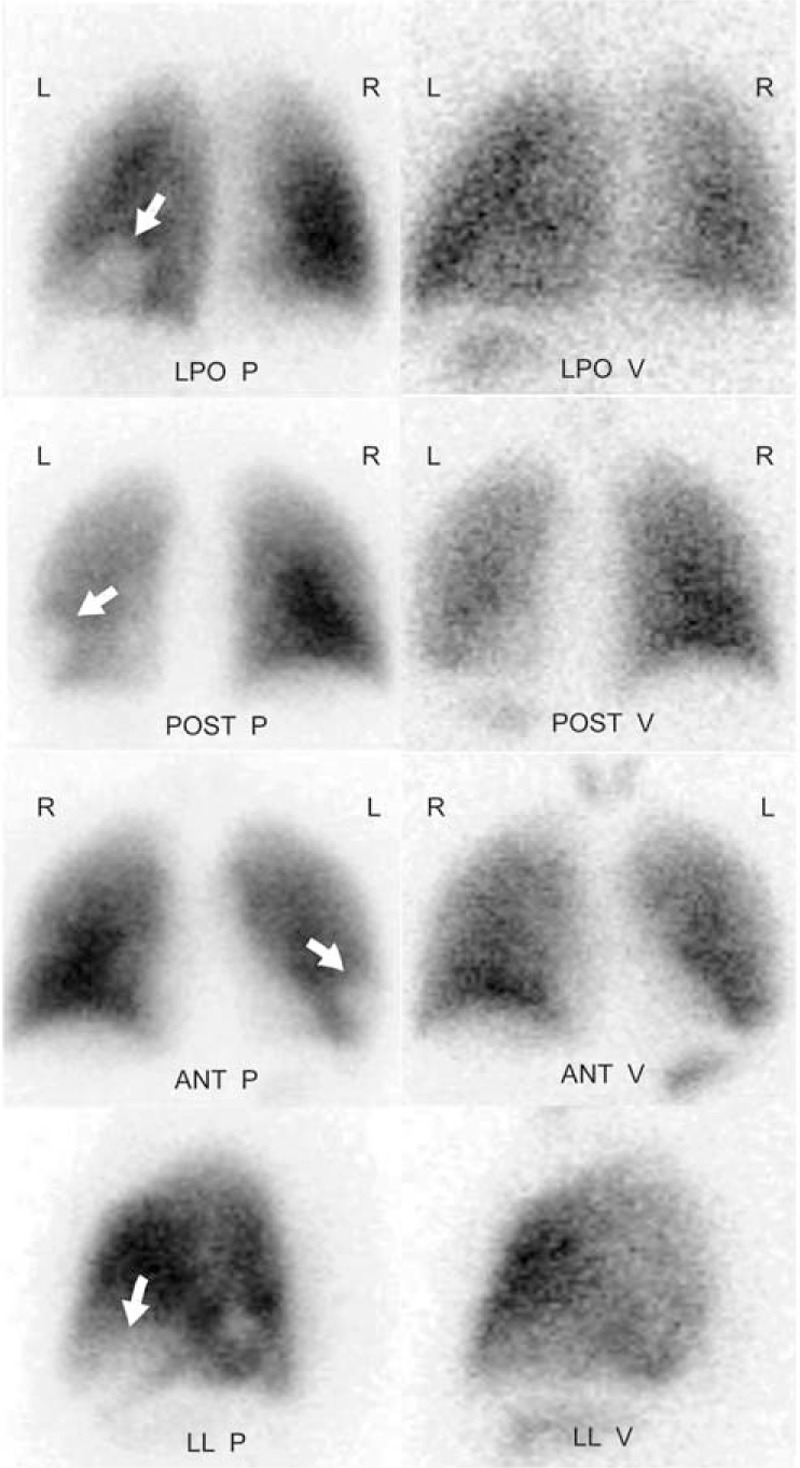
V/Q scan for PVS patient. Case 3: radionuclide V/Q scintigraphy images show a severe reduction in perfusion to the left lobe, and slight reduction in ventilation, without obvious defects. The V/Q mismatched. PVS = pulmonary vein stenosis, V/Q = ventilation–perfusion.

Only 1 person received the V/Q scan (Case 3) showed the mismatch on V/Q in the inferior lobe of the left lung (Figure 2 from Case 3).

### Echocardiography

All the patients received echocardiography. Cardiac ultrasound examinations showed pulmonary arterial hypertension in 2 persons (Table 2).

### Bronchoscope

**FIGURE 3:**
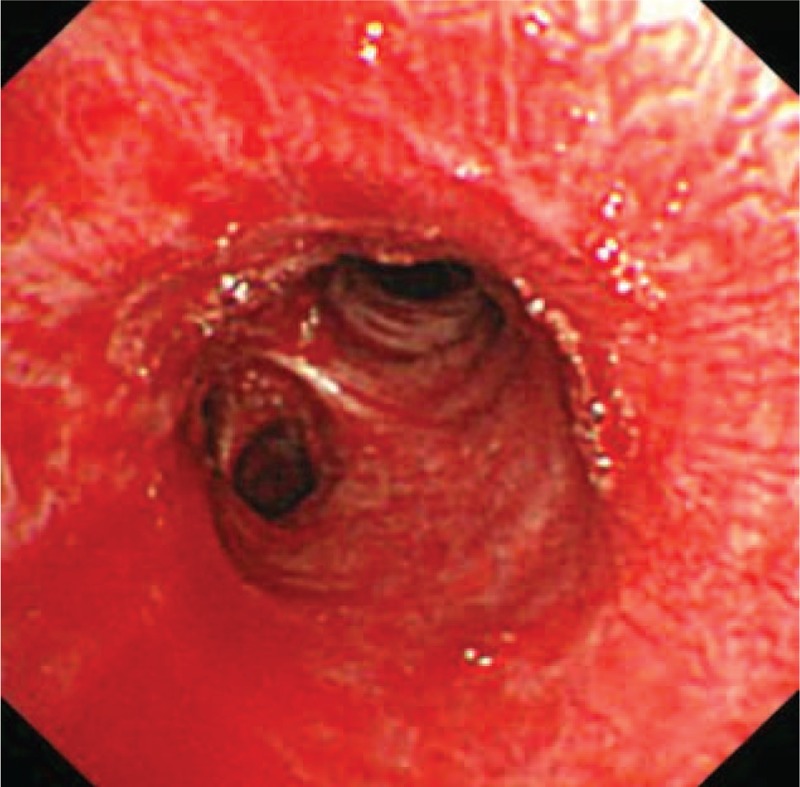
Bronchoscopy images for pulmonary vein stenosis (PVS) patient. Case 5: bronchoscopy shows congestion in the bronchial lumen mucosa and active bleeding.

Three patients underwent bronchoscope and all had congestion in the bronchial lumen mucosa, however, 1 patient had bleeding (Figure 3 from Case 5).

### Pulmonary Angiography

**FIGURE 4:**
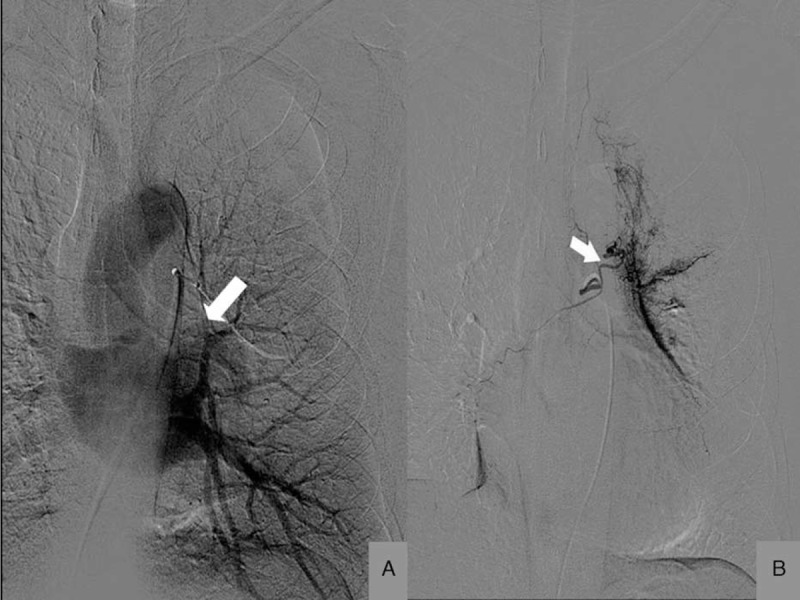
Pulmonary angiography for pulmonary vein stenosis (PVS) patient. (A) Case 5: pulmonary angiography shows an occlusion of left superior pulmonary veins. (B) Case 5: bronchial artery radiography shows that the bronchial artery was dilated.

Three patients received pulmonary angiography, which showed stenosis of the pulmonary vein. Two patients showed occlusion of pulmonary vein. Two patients received not only pulmonary arteriography but also bronchial artery radiography. Bronchial arteriography showed that bronchial artery was dilated in 2 patients who underwent selective bronchial artery embolization with gelfoam grains after bronchial artery radiography (Figure 4 from Case 5).

### Surgery and Pathological Studies

**FIGURE 5:**
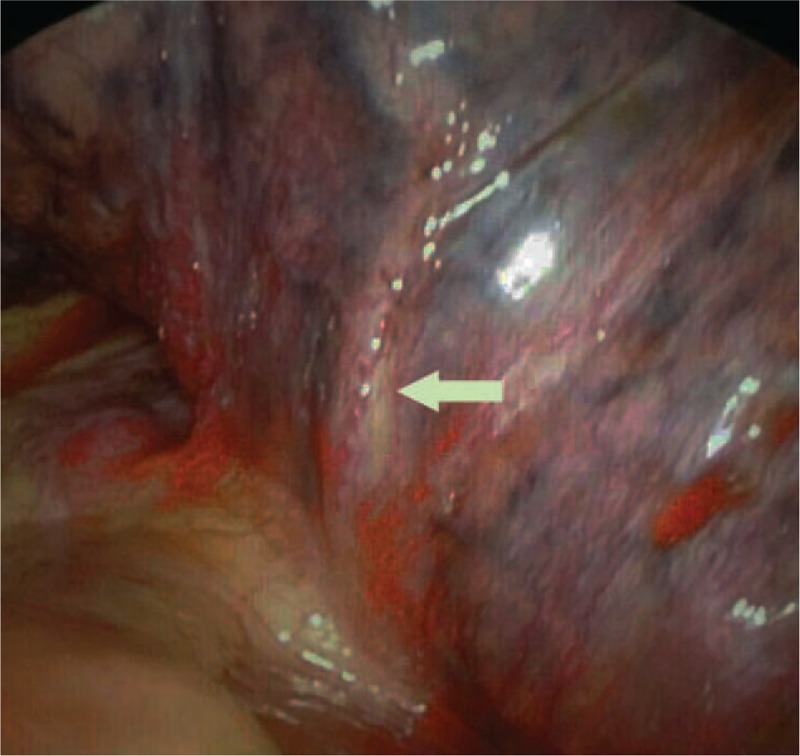
Picture in the operation for pulmonary vein stenosis (PVS) patient. A bird's-eye view of the tissue during the surgery, the arrow shows the location of the PVS that was found during the operation.

Only 1 patient underwent left upper lobe resection through video-assisted thoracoscopic biopsy, because of the suspicion of tumor. The left superior pulmonary vein occlusion and many broad patches of small vein were observed in the surgery (Figure 5 from Case 5).

**FIGURE 6:**
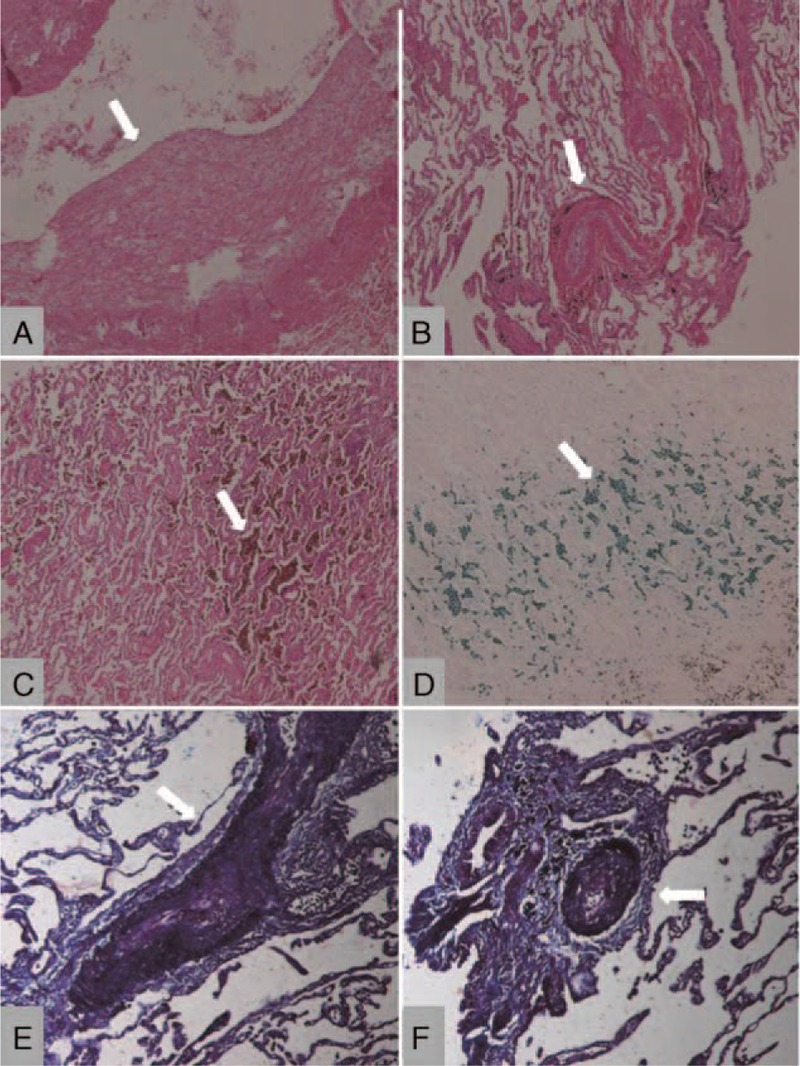
Pulmonary pathology for PVS patient. (A) Intimal hyperplasia of pulmonary vein (HE stain, original magnification 40×). (B) Pulmonary vein showing intimal fibromuscular hyperplasia, the stenosis of pulmonary vein, and the arterial wall thickening (HE stain, original magnification 40×). (C) Alveolar tissue containing numerous hemosiderin-laden macrophages, indicative of prior pulmonary congestion (HE stain, original magnification 40×). (D) Alveolar tissue containing numerous hemosiderin-laden macrophages, indicative of prior pulmonary hemorrhage (iron stain, original magnification 40×). (E) Neointimal fibromuscular hyperplasia which induces the stenosis of pulmonary vein (trichrome/elastic stain, original magnification 100×). (F) Small intrapulmonary artery, adjacent to bronchiole, showing medial hypertrophy and focal intimal hyperplasia (trichrome/elastic stain, original magnification 100×).

The histological examination of the resected left upper lobe showed intimal hyperplasia and medial thickening of large and small pulmonary veins and arteries. The intimal thickening was eccentric and involved venules and small veins in the lobular septa. The media of the veins had an increase in elastic fiber, which was confirmed by trichrome/elastic stain. The pulmonary arteries exhibited moderate to severe medial hypertrophy. The alveolar capillaries were engorged. A marked increase in hemosiderin-laden macrophages was observed within the alveoli, consistent with a past pulmonary hemorrhage (Figure 6 from Case 5).

### Prognosis

Two patients were reviewed and followed up at the outpatient department, and the other 3 were followed up via a telephone interview. All the patients were alive and only 1 patient (Case 1) complained of progressive dyspnea. The hemoptysis in 2 patients improved after bronchial artery embolization, but PVS did not change in caliber.

## DISCUSSION

The present work involved a retrospective analysis of 5 patients with PSV after radiofrequency catheter ablation, along with a literature review of published data. A number of case reports have been published so far. However, most of the reported work was from the perspective of doctors with cardiovascular expertise, and their focus was different from the current study. The purpose of this manuscript is to draw attention of not only cardiovascular but also of respiratory physicians. PVS is easily misdiagnosed by respiratory doctors due to its respiratory symptoms. Hence, the results of the present work are critical for clinical practice.

Radiofrequency catheter ablation is an efficient and effective treatment for drug-resistant AF. PVS is one of the serious complications after an AF ablation. It is defined as the reduction of >50% of pulmonary venous diameter before the ablation.^5,6^ The incidence rates range from 1.3% to 15.6% in the reported articles.^7,8^ But, the true incidence of PVS following an AF ablation is still a controversial issue. However, the lack of symptoms is the very characteristic in the patients and thus the incidence rate and severity of PVS could be underestimated.

Clinical symptoms of the patients with PVS are associated with the severity of stenosis and vascularity index of the involved vein.^7^ Mild PVS in a single vessel could be asymptomatic, which could be caused by the relatively high compensation ability of pulmonary circulation. Among symptomatic patients, what is most prevalent is the inception of exertional dyspnea, chest pain, or intermittent hemoptysis.^2,9–12^ Backflow obstruction of pulmonary veins could result in increased pulmonary capillary pressure, which could in turn induce pulmonary hypertension and right-heart failure.^4^ PVS might also dilate the supplying artery and shunt the bronchial artery and pulmonary circulation in patients with hemoptysis. The clinical symptoms of the 5 cases reported in the present study were mainly chest pain, hemoptysis, and dyspnea after exercise, which were of no specificity. Two patients were misdiagnosed for more than 1 year, which delayed the treatment, causing pulmonary arterial hypertension in these patients. The duration between radiofrequency ablation to the onset of respiratory symptoms for patients could be different. Qureshi et al^13^ reported that the median duration was 7.5 weeks. But in this group, the median duration was 5.8 months.

PVS can be diagnosed with various imaging modalities including multislice spiral chest CT angiography, magnetic resonance perfusion imaging, and catheter pulmonary venography.^14^ Physical examinations of the lungs, chest X-ray could not provide specific results for the diagnosis of PVS. Several recent studies suggested that multislice spiral chest CT angiography could help in identifying the distribution of pulmonary veins. The main imaging presentations were consolidation shadows in the lungs and pleural effusion. In a retrospective study published in 2003, Saad et al^8^ used helical CT angiography at 3, 6, and 12 months as a postprocedure and reported a complication rate for severe PVS of 18 (5%) out of 355 patients. Multislice spiral CT angiography demonstrated a left-sided predominance (83%). The main imaging presentations were the consolidation of the lungs and pleural effusion.

In addition, the symptoms of this disease and plain chest CT scanning are generally nonespecific; therefore, this disease could be easily misdiagnosed as other pulmonary diseases and has been treated with long-term antiinfective treatment, antituberculosis treatment, or even lobectomy.^8,15,16^ It is worth mentioning that most of the patients could be referred to the Department of Respiratory Medcine to seek for medical care; however, most of the respiratory physicians lack the experience in recognizing this iatrogenic disease. All the 5 patients involved in this report were misdiagnosed with pneumonia, tuberculosis, lung cancer, or PE. One patient (Case 1) was initially misdiagnosed with symptoms that a diagnosis duration of 16 months, multiple examinations, and even thoracoscopic pleural biopsy were performed in this patient. She received the long-term antiinfective and antituberculosis treatment. Awareness and early recognition of PVS may be efficient. Patients with radiofrequency catheter ablation for AF with respiratory symptoms such as shortness of breath and hemoptysis should call for a high clinical suspicion. Tests such as multislice spiral chest CT angiography would be helpful for early diagnosis, hence, should be performed regularly. Some clinicians suggest that an early intervention in the presence of a severe asymptomatic PVS leads to a clinical benefit in the long-term.^4,17^

For patients with chest pain accompanied with pleural effusion, diagnosis of PE can be made, and CT pulmonary angiography is generally performed, which cannot well display the pulmonary venous system. However, reliance on CT pulmonary angiography and V/Q scan cannot easily distinguish PVS from PE, which may confuse inexperienced pulmonary physicians and radiologists.^15,18–20^ One patient (Case 3) was suspected with PE and, thus, V/Q scan was performed; however, no pulmonary venography was performed and the patient was misdiagnosed. Therefore, differential diagnosis of these 2 diseases by carefully reviewing the medical histories (especially history of radiofrequency catheter ablation and fracture), identifying PE risk factors (oral contraceptives, varicose veins, and tumor), and performing blood examinations (including D-D dimer) should be done and evaluated comprehensively before the final diagnosis.

For patients with asymptomatic PVS, no other specific treatment except for continuing anticoagulant treatment, which could prevent thrombogenesis in pulmonary veins, is available. For patients with symptomatic PVS, an interventional therapy is generally performed to implant stent. Previous studies have shown that balloon angioplasty for PVS is safe and is associated with a good procedural success. However, high restenosis rates from 47% to 61%^2,13^ have been noted, while the restenosis rate after stenting of PVS appears to be lower, ranging from 0% to 47%.^21^ Several recent studies also reported that using drug-eluting stents such as paclitaxel-eluting stent could significantly reduce the rate of stenosis. However, large-randomized studies to demonstrate the reduced restenosis and safety of the use of drug-eluting stents in the setting of PVS are still needed.^22–25^ The treatment of symptomatic PVS is still very difficult currently. Of the 5 patients, none used implant stent, but 2 patients underwent selective bronchial artery embolization with gelfoam grains after bronchial artery radiography because of the recurrent hemoptysis. One patient with postoperative symptoms was in a stable condition in a 1-year follow-up, but PVS did not change in caliber. Only 1 patient underwent the left upper lobe resection through the video-assisted thoracoscope biopsy.

In summary, the clinical symptoms and signs of PVS after radiofrequency catheter ablation are of no specificity, and thus this disease is easily misdiagnosed. Therefore, for patients with a defined operative history, the possibility of this disease should be considered, and multislice spiral chest CT angiography and catheter examination should be performed to clarify the final diagnosis and reduce the chance of misdiagnosis. For early diagnose of this disease and to avoid misdiagnosis and mistreatment, medical history of the patients should be carefully reviewed and follow-up observations should be performed.
